# Comparison of incisive canal remodeling and root resorption in extraction vs. non-extraction fixed orthodontic retraction: a CBCT study

**DOI:** 10.3389/fphys.2025.1726454

**Published:** 2025-12-18

**Authors:** Remsh Khaled Al-Rokhami, Deguo Gao, Xiaobao Dang, Ruiqing Jiang, Guangfeng Zhang, Karim Ahmed Sakran

**Affiliations:** Zhenjiang Stomatological Hospital, Zhenjiang, Jiangsu, China

**Keywords:** bone remodeling, CBCT, incisive canal, maxillary central incisors, orthodontic retraction, root resorption

## Abstract

**Background:**

This study evaluated three-dimensional changes in incisive canal (IC) morphology, root–IC proximity, and apical root resorption following fixed orthodontic retraction, comparing extraction and non-extraction protocols.

**Methods:**

CBCT scans of 86 patients (172 maxillary central incisors; mean age 22.3 ± 5.7 years) were analyzed before (T1) and immediately after treatment (T2). Participants were assigned to extraction (n = 42) or non-extraction (n = 44) groups. Linear measurements (IC width, cortical bone width, root–IC distance, U1 length/width, IC height) were recorded at three vertical levels (H1–H3). IC and U1 volumes and surface areas were quantified using standardized 3D segmentation. Continuous group comparisons were performed using patient-averaged data, whereas incisor-level categorical outcomes were analyzed using cluster-adjusted statistical models. Root–IC proximity patterns were evaluated using Generalized Estimating Equations (GEE), and apical root resorption and volumetric changes were assessed using Linear Mixed Models (LMM). Predictors of root–IC contact/invasion and predictors of root–IC distance reduction were examined using multivariable GEE and LMM, respectively. Multiplicity was controlled using Holm–Bonferroni correction.

**Results:**

IC width and cortical bone width decreased at several levels in both groups, more prominently in extraction cases (P < 0.05). Root–IC distance decreased in all patients, with an adjusted overall mean reduction of 1.33 mm (95% CI, 1.28–1.37). LMM showed no independent effect of extraction status on root–IC distance change (B = 0.08, P = 0.079). Each millimeter of U1 retraction produced an additional 0.40 mm reduction in root–IC distance (95% CI, 0.37–0.43; P < 0.001). GEE demonstrated that each millimeter of U1 movement increased the odds of root–IC contact or invasion by 1.76-fold (95% CI, 1.21–2.56; adjusted P = 0.030). Apical root resorption was significantly higher in teeth showing canal contact or invasion, with an average 0.38 mm greater shortening compared with separated roots (95% CI, 0.08–0.69).

**Conclusion:**

Changes in root–IC proximity during orthodontic retraction are driven primarily by the magnitude of tooth movement, not extraction status. Greater retraction increases both canal approximation and the likelihood of contact/invasion, which in turn intensifies apical root resorption. Pre-treatment CBCT assessment of IC morphology and careful force and torque control are essential to minimize biomechanical overload and reduce iatrogenic risk during orthodontic retraction.

## 1 Introduction

The maxillary anterior teeth, particularly the central incisors (U1), are fundamental to facial aesthetics, phonation, and masticatory function. Their precise three-dimensional (3D) positioning is therefore critical for achieving optimal orthodontic outcomes in both functional and esthetic contexts. (Kokich, 1993; Sarver, 2001). The success of orthodontic treatment depends on understanding the anatomical boundaries that limit tooth movement within the alveolar bone. These include the cortical plates, alveolar housing, and adjacent structures such as the incisive canal (IC). (Proffit and White, 1990; Ackerman and Proffit, 1997; Cho et al., 2016).

Ackerman and Proffit’s concept of the “envelope of discrepancy” defines the biological and anatomical limits within which orthodontic and orthopedic tooth movements can be achieved. (Proffit and Ackerman, 1982; Proffit and White, 1990). Traditionally, the palatal cortical plate has been considered the principal anatomic barrier to maxillary incisor retraction, generally permitting movements of up to approximately 7 mm. (Wainwright, 1973; Ten Hoeve and Mulie, 1976; Handelman, 1996). However, from a physiological perspective, orthodontic tooth movement represents a controlled model of bone remodeling, in which mechanical forces induce balanced osteoclastic and osteoblastic activity within the alveolar and surrounding craniofacial structures. Recent evidence suggests that the incisive canal; a midline osseous structure that transmits the nasopalatine nerve and vessels between the nasal and oral cavities, may represent an additional and often underappreciated constraint. (Jacob et al., 2000; Song et al., 2009; Thakur et al., 2013).

Because of its close proximity to the roots of the maxillary central incisors, the IC has become increasingly relevant in orthodontic diagnosis and treatment planning. Excessive mechanical loading during incisor retraction may not only cause geometric proximity to the canal but also stimulate localized bone resorption and remodeling within the canal walls. Such changes can lead to undesirable outcomes such as root contact, canal invasion, or apical resorption, and occasionally to neurosensory disturbances. (Kraut and Boyden, 1998; Cho et al., 2016). Studies utilizing cone-beam computed tomography (CBCT) have demonstrated that the distance between the IC and incisor roots varies widely among individuals, and that root–canal contact after retraction is not uncommon. (Chung et al., 2015; Pan and Chen, 2019; Yu et al., 2022).

Recent CBCT studies have also demonstrated substantial variability in incisive canal morphology and root–canal spatial relationships across individuals, underscoring the need for patient-specific evaluation. (Kuc et al., 2023; Alhumaidi et al., 2024; Beshtawi, 2025). Because the canal transmits the nasopalatine neurovascular bundle, excessive root displacement or contact with the canal wall may increase the risk of sensory disturbances, reinforcing the importance of maintaining a safe root–canal distance during orthodontic retraction.

Despite advances in 3D imaging and morphometric analysis, limited data exist on the physiological remodeling of the incisive canal and surrounding bone following orthodontic treatment, particularly when comparing extraction and non-extraction protocols. As extraction therapy often involves greater mechanical loading and tooth displacement, it provides a useful model for examining the biological response of craniofacial bone under functional adaptation.

Therefore, the present study aimed to perform a three-dimensional assessment of changes in IC morphology, the spatial distance between the IC and maxillary central incisors, and the extent of root resorption following extraction and non-extraction-based fixed orthodontic therapy. By analyzing these parameters using CBCT, this research sought to elucidate how varying magnitudes of tooth movement modulate local bone remodeling, canal adaptation, and root integrity within the anterior maxilla.

## 2 Materials and methods

### 2.1 Subjects

Retrospective data were collected from archived samples of orthodontic patients treated with maxillary incisors retraction using fixed appliances (Victory Series; 3 M Unitek®, California, United States of America) between 2015 and 2022. The study is approved by a local Institutional Review Board (LZUKQ-2020–20). All participants provided written informed consent for the use of anonymized clinical and radiographic data for research purposes. The study adhered to the Declaration of Helsinki and the ALARA principle. No additional CBCT scans were obtained beyond those required for clinical diagnosis and treatment planning.

Orthodontic treatment was carried out using 0.022 × 0.028-inch Damon passive self-ligating brackets. The archwire sequence began with 0.014″Copper NiTi for initial alignment and progressed to 0.019″× 0.025″stainless steel for space closure using the sliding technique. Incisor retraction mechanics were carefully designed to respect biomechanical principles, specifically by applying controlled forces close to the center of resistance (CR) of the anterior teeth to minimize unwanted tipping, rotation, or extrusive/intrusive effects. Center of resistance location was guided by previous finite element analysis reported that CR lies approximately 6 mm apical and 4 mm posterior to the labial alveolar crest of the central incisors. (Matsui et al., 2000). Retraction was achieved using direct anchorage via mini-implants positioned between the second premolar and first molar in the maxillary arch.

The primary outcome was the change in root–IC distance. Sample size was computed using G*Power at α = 0.05 and 95% power, based on previously reported differences in root–IC distance reduction (0.5 ± 1.13 mm vs. 1.65 ± 0.98 mm). (Yu et al., 2022). Because bilateral incisors within the same patient are clustered, the power calculation was performed at the patient level, using averaged bilateral measurements to preserve statistical independence. Thus, the effective sample size matched the number of patients (not incisors). This design yielded a minimum requirement of 23 patients per group; to enhance robustness and accommodate potential exclusions, 86 eligible subjects were enrolled (non-extraction n = 44; extraction n = 42).

Analyses performed at the incisor level (e.g., proximity patterns, per-incisor logistic models) were subsequently handled using Generalized Estimating Equations (GEE) and Linear Mixed Models (LMM) to properly account for within-patient clustering. A *post hoc* check confirmed that the achieved power for the primary (patient-level) analysis remained >0.90 under the clustered data structure.

Inclusion criteria were patients diagnosed to have skeletal Class I (ANB 0°–4°) or II (ANB >4° ≤ 8°) malocclusions with mild to moderate crowding, maxillary or bimaxillary dentoalveolar protrusion, and high-quality pretreatment (T1) and post-treatment (T2, immediately after appliance removal) lateral cephalograms and cone-beam computed tomography (CBCT) images. Exclusion criteria included facial asymmetry (Menton deviation >2 mm from facial midline), midline shifts >2 mm, large diastema >2 mm, respiratory issues, signs of pre-treatment root resorption or periodontal pathology in the area of interest, missing teeth (except third molars), anomalies or history of trauma/fractures in the maxillary midline region, prior orthodontic treatment, history of systemic diseases, and any surgical interventions like corticotomy.

Subjects were grouped by premolars extraction status into a non-extraction group (typically with ≤2 mm horizontal maxillary incisor tip movement) and an extraction group (with >2 mm retraction of the maxillary incisors). Besides, the difficulty index of treatment groups was analyzed using the American Board of Orthodontics (ABO) discrepancy index (DI). (Cangialosi et al., 2004). The DI quantifies orthodontic case complexity by evaluating 10 categories: overjet, overbite, anterior/lateral open bites, crowding, occlusal relationship (Angle classification), posterior crossbites (lingual/buccal), cephalometric measurements, and other conditions (e.g., supernumerary teeth, impactions). Points are assigned based on severity, with higher totals indicating greater complexity. Besides, the maxillary sagittal position (SNA angle) and maxillary central incisors protrusion (U1-SN angle) were analyzed.

### 2.2 Analysis of IC and U1 parameters

CBCT scans were obtained using a standardized protocol (120 kVp, 5 mAs, 0.3 mm voxel size) with the KaVo Imaging System (KaVo® Dental GmbH, Bismarckring, Germany), oriented to the Frankfort horizontal plane. The field of view (FOV) was 16 × 13 cm, and the effective dose was approximately 90–110 µSv, consistent with standard orthodontic diagnostic imaging parameters. All scans were obtained as part of routine clinical records following the ALARA (As Low As Reasonably Achievable) principle, and no additional CBCT exposures were taken for research purposes.

T2 scans were voxel-based registered to T1 scans using a fully automated mutual-information algorithm (Mimics Registration Module). The registration referenced the anterior cranial base (bounded anteriorly by the cribriform plate, posteriorly by the dorsum sellae, and laterally by the orbital roofs) and the palatal vault (bounded anteriorly by the anterior nasal spine and posteriorly by the posterior nasal spine) as stable anatomical structures. This reference framework has been validated in previous CBCT superimposition study. (Chung et al., 2021). The mutual-information algorithm was applied with standard settings: 1,000 maximum iterations, 64 histogram bins, and 20% random voxel sampling. Convergence was defined as a change in mutual information <0.001 between successive iterations. Internal validation was performed by re-registering 10 randomly selected cases, and registration errors were calculated as the Euclidean distance between corresponding landmarks in T1 and T2 scans. The mean registration error was 0.27 ± 0.04 mm, confirming high reproducibility and consistency with accepted craniofacial superimposition accuracy thresholds (<0.3 mm).

To ensure consistency in measurement levels between pre- and post-treatment scans, maintain uniform biomechanical conditions, and minimize confounding effects of uncontrolled tipping or torque, only cases exhibiting primarily bodily movement of the maxillary incisors were included, as verified by retraction mechanics and superimposed CBCT evaluation. Bodily movement was confirmed by sagittal superimposition showing less than 5° change in U1–SN inclination and minimal change in root–crown angulation, confirming translation-dominant movement rather than tipping. The mean change in U1–SN angle was 2.3° ± 1.9°.

Then, U1 movement is calculated as the distance between central incisor edges measured through tooth long axis, referenced to the Frankfort horizontal plane.

**FIGURE 1:**
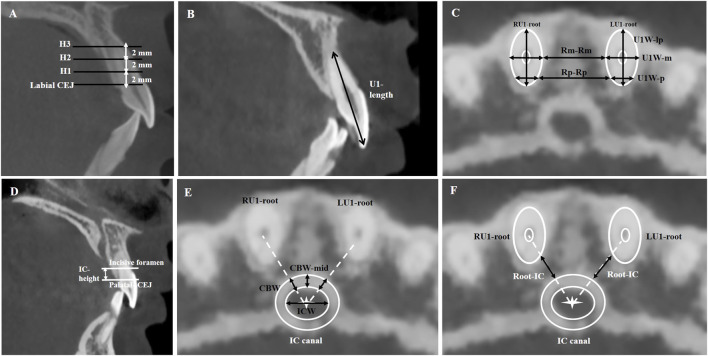
Analysis of incisive canal (IC) and maxillary central incisor (U1) parameters. **(A)** Schematic representation of measurement levels positioned at three vertical heights above the labial cementoenamel junction (CEJ): H1 (2 mm), H2 (4 mm), and H3 (6 mm). **(B)** U1 length was measured from the incisal edge to the root apex along the sagittal plane. **(C)** Root morphology quantification: (1) U1 root widths at medial (U1W-m), posterior (U1W-p), and labial-palatal (U1W-lp) points; (2) Inter-root distances measured between medial points (Rm-Rm) and posterior points (Rp-Rp) of adjacent roots. **(D)** IC height is the vertical distance from the incisive foramen plane to the palatal cementoenamel junction (PCEJ). **(E)** IC and cortical bone measurements: (1) IC width (ICW) was defined as the maximum canal diameter at the IC center (IC-Cen, marked by white star), (2) Cortical bone width (CBW) around IC was measured along the axis connecting IC-Cen to the adjacent root center (dotted white line), (3) CBW-mid represents the cortical bone thickness in the midsagittal plane. **(F)** The root-IC distance was the shortest distance between the lateral boundary of U1 root and the lateral IC cortical border and was measured along the axis (dotted white line) connecting each root center to the IC center (marked by white star).

Linear measurements of the central incisor and the incisive canal were obtained using *Invivo* Dental Imaging Software (version 6, Anatomage, San Jose, CA) through axial views of the maxillary anterior region at three heights above the labial cementoenamel junction of the maxillary central incisor (2 mm, 4 mm, and 6 mm; H1, H2, and H3, respectively), as shown in Figure 1A. (Al-Rokhami et al., 2022; Al-Rokhami et al., 2025; Yu et al., 2022).

The length of the U1 was measured from the crown tip to the root tip along the tooth’s long axis in the sagittal plane (Figure 1B). The width of the U1 root was measured along the mesiodistal aspect at the most medial (U1W-m) and posterior (U1W-p) points, as well as along the labiopalatal aspect (U1W-lp) (Figure 1C). The inter-root distance was measured at the most medial (Rm-Rm) and posterior (Rp-Rp) points of the U1 root (Figure 1C). Then, root resorption was assessed by measuring changes in the incisor’s length and width between pre-treatment and post-treatment scans (Supplementary Figure S1).

Incisive canal height was defined as the vertical distance from the incisive foramen plane (the horizontal plane passing through the lowest point of the IC’s buccal wall) to the palatal cementoenamel junction (PCEJ) (Figure 1D). As presented in Figure 1E, the incisive canal width (ICW) was defined as the maximum width across the center of the clearly visible radiolucent lumen of the IC (IC-Cen; marked by a white star). Measurements of cortical bone width (CBW) was taken along a line (marked by a dotted white line) connecting IC-Cen and the center of the incisor root. Additionally, cortical bone width in the midsagittal plane (CBW-mid), passing through IC-Cen, was measured.

The root-IC distance was the shortest distance between the lateral boundary of the maxillary central root and the lateral IC cortical border. The root-IC distance was taken along a line (marked by a dotted white line) connecting IC-Cen and the center of the incisor root (Figure 1F).

Volumetric analyses of the IC and U1 were performed using 3D segmentation software (Mimics 21.0, Materialise, Leuven, Belgium). All segmentations were performed using gray-value thresholds. For tooth segmentation, the initial threshold was based on enamel–dentin contrast and refined to a gray-value range of 1,400–3,071 to isolate the U1 root from surrounding bone (Supplementary Figure S2A). For the incisive canal, segmentation was guided by cortical/trabecular contrast and refined to a gray-value range of 555–1988, effectively delineating the canal lumen from adjacent osseous structures (Supplementary Figure S2B). Region-growing was used to delineate U1 and IC boundaries, followed by manual contour refinement and 3D surface smoothing (iteration = 2, smoothing factor = 0.6) to minimize artifacts while preserving anatomical accuracy.

**FIGURE 2:**
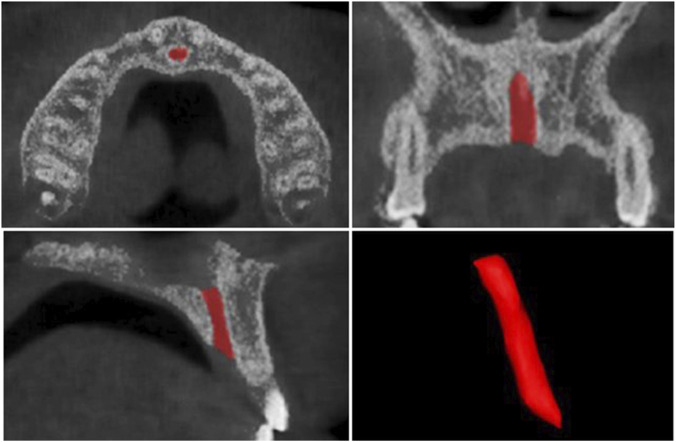
Segmentation of the internal portion of the incisive canal and the resulting 3D model used for volumetric measurement.

**FIGURE 3:**
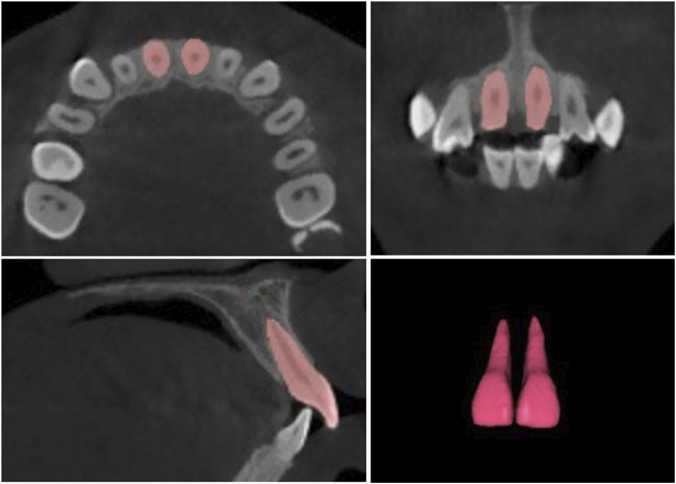
Segmentation of the maxillary central incisors and the resulting 3D model used for volumetric measurement.

The IC volume was segmented superiorly at the nasal cavity floor and inferiorly at the palatal roof (Figure 2), while the U1 was segmented from the incisal ridge to the root apex (Figure 3). The resulting 3D reconstructions were used to automatically calculate volumes and surface areas for both structures. Volumetric segmentations were evaluated for reliability using intraclass correlation coefficients (ICCs), yielding intra- and inter-examiner ICCs of 0.97 and 0.92 for U1 and 0.97 and 0.90 for IC, respectively, confirming excellent reproducibility.

Root-IC proximity patterns were classified as separation (Supplementary Figure S3), approximation (Supplementary Figure S4), contact (Supplementary Figure S5), or invasion (Supplementary Figure S6), based on comparisons of root-IC distance between T1 and T2 scans. If the root contacted the IC’s lateral cortical boundary, the root-IC distance was recorded as zero. When the root partially invaded the cortical bone without reaching the lumen, this invasion distance was recorded as a negative root-IC distance, and the remaining CBW was measured. Full root invasion into the IC lumen resulted in a negative root-IC distance, with CBW recorded as zero. Further, based on T2-T1 calculation, a positive root-IC distance indicated separation, while approximation, contact, and invasion were represented by negative values.

### 2.3 Statistical analysis

All analyses were performed in SPSS version 25.0. Data distribution was assessed using the Shapiro–Wilk test and found to be non-normal; therefore, non-parametric or generalized/linear mixed models were used as appropriate. Statistical significance was set at P < 0.05.

For continuous linear and volumetric variables, bilateral measurements were averaged per patient to preserve independence at the patient level. Intra-group differences were assessed using Wilcoxon ranks paired test while inter-group differences (extraction vs. non-extraction protocols) were tested using the Mann–Whitney U test. Because each outcome was evaluated at three vertical levels (H1–H3), multiplicity was controlled using the Holm–Bonferroni correction across the three comparisons per variable.

Root–IC proximity patterns were analyzed at the incisor level because proximity events may differ between right and left sides. Differences in pattern distribution between treatment groups across the three vertical levels were assessed using Generalized Estimating Equations (GEE) with a binomial distribution and an exchangeable working correlation matrix. Patient ID was specified as the clustering variable. Holm–Bonferroni correction was applied across the three levels.

To compare apical root resorption across proximity patterns, a Linear Mixed Model (LMM) with a random intercept for patient was used, accounting for clustering of bilateral incisors. Pairwise differences were evaluated using Dunn’s *post hoc* test, with Holm–Bonferroni adjustment across proximity patterns at each level.

Initial comparisons of changes (T2–T1) in volumes and surface areas across proximity patterns were conducted using the Kruskal–Wallis test with Dunn’s *post hoc* procedure. To account for within-patient clustering, these analyses were complemented by Linear Mixed Models with a random intercept for patient. Holm–Bonferroni correction was applied across proximity patterns at each level. This combined analytic strategy allowed patient-level inference for quantitative variables while preserving side-specific information when clinically meaningful. This approach aligns with recommended strategies for handling clustered or bilateral data in dental and craniofacial research. (Park et al., 2010; Koletsi et al., 2012; Seehra and Pandis, 2024).

Univariate and multivariate GEE binary logistic regression models were applied to identify factors associated with negative U1–IC distance (contact or invasion). Clustering by patient was handled using an exchangeable correlation structure. Eighteen univariate GEE models (one per factor) were run, and P-values were adjusted using the Holm–Bonferroni method to control family-wise error. Variables with adjusted P < 0.05 or strong clinical relevance were included in the multivariate GEE model to determine independent predictors while estimating the adjusted effect of extraction.

A Linear Mixed Model with a random patient intercept was used to evaluate predictors of root–IC distance reduction (T2–T1), accommodating bilateral clustering. All candidate predictors (extraction status, sex, age, treatment duration, U1 movement, baseline IC height, baseline root–IC distance, and ABO discrepancy index) were entered into a full multivariable model. Fixed-effect coefficients (B), standard errors, and 95% confidence intervals were reported. Holm–Bonferroni–adjusted P-values were calculated across the eight predictors.

Two calibrated examiners performed all measurements after 8 h of structured training using 20 practice CBCT scans. Intra-examiner repeatability, assessed by re-measuring 15% of cases after 2 weeks, demonstrated excellent reliability (ICC = 0.90–0.97). Inter-examiner agreement was similarly strong (ICC = 0.84–0.96). Examiners were blinded to treatment group and time point.

To ensure treatment-related changes exceeded CBCT measurement error, repeatability for U1 length and root–IC distance was further evaluated (15% of cases, repeated after 2 weeks). With a 0.3-mm voxel size, repeated-measure SD ranged from 0.15 to 0.17 mm, and the MDC_95_ ranged from 0.42 to 0.47 mm. Bland–Altman analysis showed minimal bias (0.21–0.30 mm) and narrow limits of agreement (−0.113–0.594 mm). Observed treatment-related changes (0.7–1.0 mm) exceeded these thresholds, confirming that detected differences reflected true biological remodeling rather than voxel-level noise.

A biostatistician with expertise in clustered data analysis independently reviewed the statistical workflow, including the implementation of GEE and LMM models. All multiplicity adjustments were performed using the Holm–Bonferroni method applied across each prespecified family of tests.

## 3 Results

### 3.1 Descriptive data

The study included 86 participants (mean age = 22.28 ± 5.74 years; 36 males, 50 females) treated with fixed orthodontic appliances. Participants were divided into a non-extraction group (n = 44) and an extraction group (n = 42). Demographic data and ABO discrepancy index scores are presented in the Supplementary Table S1.

### 3.2 Morphological changes of IC and U1

Linear measurements showed a post-treatment decrease in the IC width at all levels (H1–H3) in both groups, with a greater reduction in the extraction group. The decrease was significant at H1 in the non-extraction group (P < 0.001) and at H1 and H2 in the extraction group (P < 0.001 and P = 0.029, respectively). However, after applying the Holm-Bonferroni correction, the significant reductions between the groups at H1 and H2 became non-significant trends (P = 0.087) (Table 1).

**TABLE 1:** Comparison of incisive canal and maxillary central incisor linear measurements before (T1) and after (T2) treatment between treatment groups.

Measure, mm	Non-extraction			Extraction			P value^a^	Adjusted P value
	T1	T2	P value^b^	T1	T2	P value^b^		
IC width, mm								
H1	4.04 ± 0.68	3.76 ± 0.63	<0.001*	3.72 ± 0.89	3.10 ± 0.76	<0.001*	0.029*	0.087
H2	3.48 ± 0.69	3.45 ± 0.76	0.134	3.34 ± 0.89	3.05 ± 0.55	0.029*	0.034*	0.087
H3	3.35 ± 0.84	3.36 ± 0.74	0.696	3.01 ± 0.78	3.31 ± 0.96	0.289	0.852	0.852
IC cortical bone width (CBW), mm								
H1	0.91 ± 0.22	0.72 ± 0.35	0.001*	0.83 ± 0.26	0.57 ± 0.44	0.012*	0.818	0.974
H2	0.93 ± 0.17	0.85 ± 0.30	0.160	0.89 ± 0.13	0.69 ± 0.31	0.001*	0.176	0.528
H3	0.87 ± 0.15	0.82 ± 0.24	0.292	0.91 ± 0.26	0.85 ± 0.11	0.998	0.487	0.974
IC cortical bone width-mid (CBW-mid), mm								
H1	0.94 ± 0.29	0.95 ± 0.18	0.676	0.96 ± 0.17	0.83 ± 0.18	0.028*	0.037*	0.074
H2	0.92 ± 0.24	0.92 ± 0.29	0.399	0.85 ± 0.14	0.88 ± 0.14	0.605	0.437	0.437
H3	0.80 ± 0.16	0.95 ± 0.24	<0.001*	0.97 ± 0.22	0.86 ± 0.19	0.006*	<0.001*	<0.001*
U1 root-IC distance, mm								
H1	1.53 ± 0.65	0.80 ± 0.71	<0.001*	1.71 ± 0.54	−0.01 ± 0.50	<0.001*	<0.001*	<0.001*
H2	1.96 ± 0.70	1.08 ± 0.78	<0.001*	2.15 ± 0.72	0.61 ± 0.82	<0.001*	0.016*	0.016*
H3	2.58 ± 1.00	1.85 ± 1.37	<0.001*	2.75 ± 0.71	1.20 ± 1.21	<0.001*	0.002*	0.004*
**U1 length, mm**	21.50 ± 1.35	20.49 ± 1.41	<0.001*	21.16 ± 1.33	19.14 ± 1.96	<0.001*	0.005*	
U1 width, mm								
H1	4.09 ± 0.49	3.99 ± 0.31	0.224	3.86 ± 0.42	3.68 ± 0.50	0.106	0.395	0.395
H2	3.22 ± 0.42	3.08 ± 0.55	0.012*	3.09 ± 0.45	2.45 ± 1.02	<0.001*	0.005*	0.015*
H3	1.89 ± 0.28	0.98 ± 1.04	<0.001*	1.89 ± 0.23	0.74 ± 1.07	<0.001*	0.074	0.148

Data are reported in millimeters (mm) by mean ± standard deviation.

^a^ Mann-Whitney U test comparing changes (T2-T1) between the two groups. Adjusted p-values were calculated using the Holm–Bonferroni method across the three measurement levels (H1–H3) for each outcome. Analyses were performed at the patient level using averaged bilateral measurements to preserve statistical independence.

^b^ Wilcoxon ranks paired test comparing T1 and T2 within groups.

* Significant P-values.

Cortical bone width (CBW) decreased significantly at H1 in the non-extraction group (P = 0.001) and at both H1 and H2 in the extraction group (P = 0.012 and P = 0.001, respectively). Interestingly, CBW-mid increased at H3 in the non-extraction group (P < 0.001) but decreased at H1 and H3 in the extraction group (P = 0.028 and P = 0.006, respectively), suggesting site-specific cortical responses to tooth movement (Table 1).

**FIGURE 4:**
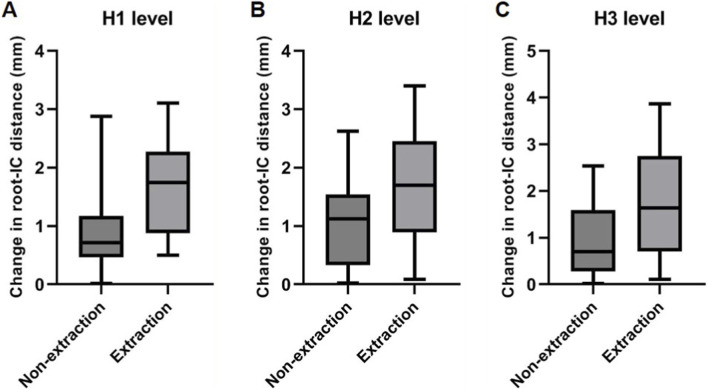
Box plots illustrating the distribution and mean shift in changes of root–IC distance between extraction and non-extraction groups at three measurement levels: **(A)** H1, **(B)** H2, and **(C)** H3.

The U1 root–IC distance decreased in both groups (Table 1; Figure 4), more prominently in the extraction cases (H1: 1.71 ± 0.78 mm; 95% CI, 1.47–1.96 vs. 0.73 ± 0.86 mm; 95% CI, 0.47–0.99; P < 0.001; H2: 1.54 ± 1.09 mm; 95% CI, 1.20–1.88 vs. 0.88 ± 0.94 mm; 95% CI, 0.59–1.16; P = 0.016; H3: 1.55 ± 1.29 mm; 95% CI, 1.15–1.96 vs. 0.72 ± 0.92 mm; 95% CI, 0.44–1.00; P = 0.004). U1 root length decreased significantly in both groups, with greater apical root resorption observed in the extraction group (2.02 ± 1.71 mm; 95% CI, 1.49–2.56) compared with the non-extraction group (1.01 ± 0.74 mm; 95% CI, 0.78–1.23; P = 0.005). A significant post-treatment reduction in U1 width was also observed, particularly at H2 in the extraction group (P = 0.015).

Volumetric and surface area analyses confirmed these trends: both U1 volume and surface area decreased significantly in both groups, with greater reductions in the extraction group (U1 volume: 28.78 ± 28.49 mm^3^; 95% CI, 22.59–34.96 vs. 15.05 ± 19.01 mm^3^; 95% CI, 11.03–19.08; P < 0.001; U1 surface area: 21.87 ± 19.54 mm^2^; 95% CI, 17.63–26.11 vs. 10.29 ± 8.87 mm^2^; 95% CI, 8.41–12.17; P < 0.001). IC volume reduction was notably higher in extraction cases (40.15 ± 31.65 mm^3^; 95% CI, 30.29–50.02 vs. 27.48 ± 21.98 mm^3^; 95% CI, 20.79–34.16; P = 0.033), indicating that orthodontic retraction induces measurable three-dimensional remodeling of both the incisive canal and adjacent tooth structures (Table 2).

**TABLE 2:** Comparison of incisive canal and central incisor volumes and surface areas at T1 and T2 between treatment groups.

Parameter	Non-extraction			Extraction			P value^a^
	T1	T2	P value^b^	T1	T2	P value^b^	
IC volume, mm^3^	123.34 ± 64.42	120.46 ± 47.95	0.592	116.31 ± 63.98	108.73 ± 55.30	0.409	0.033*
IC area, mm^2^	170.11 ± 55.21	168.52 ± 41.59	0.758	156.62 ± 47.36	154.98 ± 46.69	0.796	0.566
U1 volume, mm^3^	516.23 ± 57.32	511.01 ± 56.36	0.042*	476.07 ± 55.52	451.62 ± 61.31	<0.001*	<0.001*
U1 area, mm^2^	416.28 ± 31.06	410.13 ± 30.32	<0.001*	394.13 ± 31.51	375.06 ± 38.24	<0.001*	<0.001*

Data are reported either in square millimeters (mm^2^) or cubic millimeters (mm^3^) and presented by mean ± standard deviation.

^a^ Mann-Whitney U test comparing changes (T2-T1) between the two groups.

^b^ Wilcoxon ranks paired test comparing T1 vs. T2 within group.

* Significant P-values.

Analyses were performed at the patient level using averaged bilateral measurements to preserve statistical independence.

### 3.3 Patterns of Root-IC proximity and morphological changes

**FIGURE 5:**
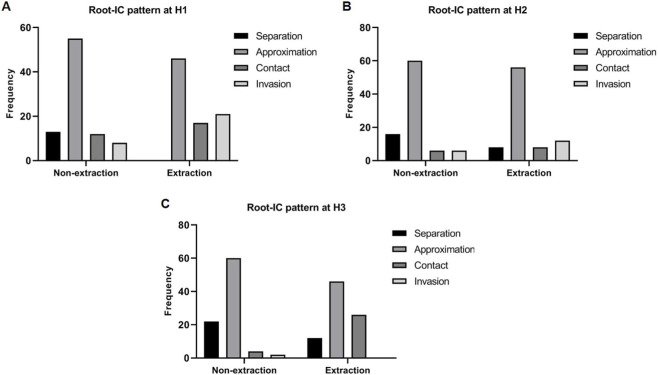
Stacked bar charts showing the relative frequencies of root–IC proximity patterns (separation, approximation, contact, and invasion) at post-treatment across extraction and non-extraction groups for each measurement level: **(A)** H1, **(B)** H2, and **(C)** H3.

Among the 172 central incisors analyzed, root–IC proximity patterns showed 13.8% separation, 62.6% approximation, 14.1% contact, and 9.5% invasion. After adjustment for clustering in the GEE model, proximity distributions differed significantly between groups, with significantly more invasion and/or contact patterns observed in extraction cases at H1 (OR = 4.27, 95% CI 2.03–9.01, P < 0.001) at H3 (OR = 4.17, 95% CI 1.77–9.80, P = 0.002) (Table 3; Figure 5).

**TABLE 3:** Comparison of root–IC proximity patterns at T2 between extraction and non-extraction groups across H1, H2, and H3 levels using a generalized estimating equation model.

Root-IC proximity	Non-extraction (n = 88 centrals)	Extraction (n = 84 centrals)	SE	OR (95% CI)	P value	Adjusted P value
H1, n (%)			0.38	4.27 (2.03–9.01)	<0.001*	<0.001*
Separation	13 (14.8)	0 (0)				
Approximation	55 (62.5)	46 (54.8)				
Contact	12 (13.6)	17 (20.2)				
Invasion	8 (9.1)	21 (25)				
H2, n (%)			0.41	2.04 (0.90–4.63)	0.087	0.087
Separation	16 (18.2)	8 (9.5)				
Approximation	60 (68.2)	56 (66.7)				
Contact	6 (6.8)	8 (9.5)				
Invasion	6 (6.8)	12 (14.3)				
H3, n (%)			0.43	4.17 (1.77–9.80)	0.001*	0.002*
Separation	22 (25)	12 (14.3)				
Approximation	60 (68.2)	46 (54.8)				
Contact	4 (4.5)	26 (31)				
Invasion	2 (2.3)	0 (0)				

Data are reported by frequency and rate, n (%).

SE, standard error; OR, odds ratio; CI, confidence interval.

P values derived from a generalized estimating equations (GEE) binomial model with an exchangeable correlation structure, accounting for within-patient clustering of bilateral incisors (inference at the patient level).

Adjusted P values were calculated using the Holm–Bonferroni correction for the three measurement levels.

* Significant P-values.

Linear mixed-model analysis accounting for within-subject clustering showed that invasion patterns were associated with the greatest apical root resorption, particularly at H1 and H2 (Table 4). At H1, estimated mean resorption for invasion was 2.45 mm (95% CI 2.07–2.83), while separation and approximation exhibited significantly lower resorption (estimated means 0.83 mm and 0.91 mm, respectively; both P < 0.001). Contact at H1 did not differ significantly from invasion (P = 0.249). At H2, invasion again demonstrated higher resorption (2.78 mm; 95% CI 2.17–3.39) relative to separation and approximation (both P < 0.001), while contact showed a non-significant trend toward higher resorption (P = 0.079).

**TABLE 4:** Linear mixed model comparison of apical root resorption (172 maxillary central incisors) among different root–IC proximity patterns at T2.

Level	Root-IC proximity	Root resorption, mm	Adjusted mean (95% CI), mm	SE	Raw P value	Adjusted P value
H1	Separation	0.84 ± 0.41^a^	0.83 (0.25–1.40)	0.29	<0.001*	<0.001*
	Approximation	0.90 ± 0.89^a^	0.91 (0.72–1.11)	0.10	<0.001*	<0.001*
	Contact	2.13 ± 1.02^b^	2.14 (1.76–2.51)	0.19	0.249	0.249
	Invasion (ref)	2.52 ± 1.59^b^	2.45 (2.07–2.83)	0.19		
H2	Separation	1.10 ± 0.95^a^	1.10 (0.91–1.30)	0.10	<0.001*	<0.001*
	Approximation	1.31 ± 1.24^a^	1.20 (0.73–1.67)	0.24	<0.001*	<0.001*
	Contact	2.07 ± 1.02^b^	2.08 (1.58–2.57)	0.25	0.079	0.079
	Invasion (ref)	2.87 ± 2.09^b^	2.78 (2.17–3.39)	0.31		
H3	Separation	1.12 ± 0.86	0.98 (0.59–1.38)	0.20	0.383	1.000
	Approximation	1.29 ± 1.10	1.30 (1.08–1.53)	0.11	0.575	1.000
	Contact	1.95 ± 1.82	1.85 (1.43–2.27)	0.21	0.992	1.000
	Invasion (ref)	1.86 ± 0.00	1.86 (0.09–3.81)	0.98		

Root resorption is reported in millimeters (mm). Values are presented as mean ± standard deviation; adjusted means are shown with 95% confidence intervals.

Raw P-values were derived from Linear Mixed Models (LMM) with a random intercept for patient, accounting for clustering of bilateral incisors. Adjusted P-values were calculated using the Holm–Bonferroni method for proximity-pattern comparisons at each level. Invasion pattern served as the reference category for each vertical level (H1–H3).

Pairwise differences were assessed using Dunn’s *post hoc* test; identical superscript letters (a, b) indicate non-significant differences.

* Significant P-values.

Volumetric and surface-area analyses (Table 5; Supplementary Table S2) demonstrated that root–IC proximity significantly influenced three-dimensional remodeling of both the IC and the U1. The Linear Mixed Model, accounting for within-subject clustering and Holm–Bonferroni correction, revealed that at H1, reductions in IC volume and area were significantly greater in invasion cases than in the other proximity patterns (adjusted P < 0.05). Approximation and contact groups also showed intermediate but trends reductions relative to separation. At H3, IC volume reduction remained significantly greater in closer proximity categories (unadjusted P = 0.032), although this difference did not persist after multiple-comparison adjustment. Changes in U1 volume and surface area followed the same directional trend, larger reductions with closer proximity, but did not reach significance after adjustment. Collectively, these results indicate that root–IC contact and invasion are associated with enhanced three-dimensional bone remodeling, particularly of the IC walls in the coronal and apical regions, consistent with localized adaptive resorption and deposition under orthodontic loading.

**TABLE 5:** Comparison of changes (T2-T1) in incisive canal and central incisor volumes and surface areas as per root-IC proximity pattern.

Level	Root-IC proximity	IC volume, mm^3^	IC area, mm^2^	U1 volume, mm^3^	U1 area, mm^2^
H1	Separation (n = 13)	22.04 ± 22.42^a^	20.96 ± 22.50^a^	13.89 ± 15.38	8.21 ± 10.41^a^
	Approximation (n = 101)	23.74 ± 19.28^ab^	21.93 ± 17.26^ab^	20.55 ± 22.90	14.38 ± 13.43^ab^
	Contact (n = 29)	26.99 ± 26.59^bc^	24.19 ± 16.21^ac^	22.32 ± 22.80	19.31 ± 15.54^b^
	Invasion (n = 29)	40.72 ± 29.20^c^	34.00 ± 24.96^c^	28.91 ± 35.17	21.50 ± 23.87^b^
	Raw P value	0.001*	0.014*	0.273	0.033*
H2	Separation (n = 24)	23.90 ± 20.33	22.11 ± 20.03	19.67 ± 18.14	12.03 ± 13.15
	Approximation (n = 116)	23.85 ± 27.42	25.06 ± 17.85	20.54 ± 23.96	15.04 ± 14.74
	Contact (n = 14)	28.61 ± 22.45	28.59 ± 21.31	27.46 ± 38.60	19.98 ± 25.49
	Invasion (n = 18)	37.42 ± 28.82	31.11 ± 24.06	27.91 ± 26.19	23.86 ± 17.29
	Raw P value	0.072	0.394	0.524	0.070
H3	Separation (n = 34)	20.14 ± 18.91^a^	19.45 ± 15.36^a^	20.31 ± 23.96	14.95 ± 13.84
	Approximation (n = 106)	33.81 ± 27.23^b^	28.58 ± 22.37^b^	20.57 ± 25.15	15.33 ± 17.30
	Contact (n = 30)	43.69 ± 31.23^b^	39.64 ± 26.55^c^	27.04 ± 28.61	19.23 ± 14.78
	Invasion (n = 2)	58.81 ± 0.00^b^	41.70 ± 0.00^abc^	26.20 ± 0.00	16.21 ± 0.00
	Raw P value	0.003*	0.004*	0.575	0.677

Data are reported either in square millimeters (mm^2^) or cubic millimeters (mm^3^) and presented by mean ± standard deviation.

Raw *P*-value calculated by Kruskal–Wallis test, followed by Dunn’s *post hoc* test. Identical superscript letters (a, b, etc.) denote non-significant differences between categories.

* Significant *P*-values.

Linear Mixed Model of changes in incisive canal and central incisor volumes and surface areas is reported in Supplementary Table S2.

### 3.4 Factors contributing to closer U1-IC proximity

Across all evaluated incisors and measurement levels (H1–H3), positive U1–IC distances (separation) were recorded 71 times, and negative distances (approximation, contact, or invasion) 445 times. Univariate GEE analysis (clustered by patient) identified extraction protocol, treatment duration, U1 movement magnitude, U1 length, U1 volumetric measures, IC height, and ABO index score as significant predictors of root–IC contact or invasion (P < 0.05). After Holm–Bonferroni adjustment for multiple comparisons, only treatment duration (adjusted P = 0.014), U1 movement (adjusted P < 0.001), U1 volumetric measures (adjusted P < 0.001), and IC height (adjusted P < 0.001) remained significant (Table 6).

**TABLE 6:** Generalized estimating equations–based univariate and multivariate logistic regression analysis of factors associated with a negative U1–IC distance (root proximity, contact, or invasion into the IC).

Factor	U1-IC distance		Univariate analysis		Multivariate analysis		
	Positive (71)	Negative (445)	Raw P-value	Adjust P-value	SE	OR (95% CI)	Raw P-value
Extraction, n (%)			0.005*	0.065	0.46	1.24 (0.50–3.11)	0.645
Yes	20 (7.9)	232 (92.1)					
No	51 (19.3)	213 (80.7)					
Sex, n (%)			0.086	0.774	0.33	0.58 (0.30–1.12)	0.104
Male	21 (9.7)	195 (90.3)					
Female	50 (16.7)	250 (83.3)					
Age, y	21.47 ± 5.52	22.42 ± 5.74	0.468	1.000	0.03	0.99 (0.93–1.06)	0.767
Treatment duration, y	2.41 ± 0.99	3.04 ± 1.06	0.001*	0.014*	0.13	1.31 (0.99–1.72)	0.051
U1 movement, mm	−0.27 ± 1.53	−2.25 ± 2.15	<0.001*	<0.001*	0.19	1.76 (1.21–2.56)	0.003*
U1 length, mm	21.12 ± 1.24	21.70 ± 1.30	0.027*	0.324	0.22	0.88 (0.56–1.38)	0.577
U1 volume, mm^3^	494.83 ± 60.15	507.83 ± 55.57	<0.001*	<0.001*	0.03	0.99 (0.94–1.04)	0.771
U1 area, mm^2^	404.40 ± 33.31	412.13 ± 30.63	<0.001*	<0.001*	0.04	1.00 (0.91–1.11)	0.946
Rm-rm distance, mm	4.45 ± 0.82	4.29 ± 0.76	0.058	0.580			
Rp-rp distance, mm	5.19 ± 0.78	4.95 ± 0.71	0.347	1.000			
IC height, mm	5.04 ± 1.03	4.23 ± 1.18	<0.001*	<0.001*	0.17	0.67 (0.48–0.95)	0.022*
IC width, mm	3.55 ± 0.68	3.49 ± 0.71	0.174	1.000			
IC volume, mm^3^	122.08 ± 61.95	115.83 ± 59.57	0.718	1.000			
IC area, mm^2^	164.63 ± 53.53	163.34 ± 51.13	0.986	1.000			
Root-IC distance, mm	2.08 ± 0.79	2.29 ± 0.65	0.532	1.000	0.26	0.76 (0.45–1.29)	0.311
ABO index score, points	10.43 ± 6.22	13.31 ± 7.56	0.036*	0.396	0.02	1.03 (0.98–1.09)	0.188
SNA (°)	83.16 ± 3.50	82.81 ± 3.03	0.279	1.000			
U1-SN (°)	103.53 ± 8.42	103.62 ± 12.18	0.637	1.000			

All continuous data were reported by mean ± standard deviation, in years (y), millimeters (mm), square millimeters (mm2), cubic millimeters (mm3), points, or degrees (°); extraction and sex reported by frequency and rate, n (%).

SE, standard error; OR, odds ratio; CI, confidence interval. All raw P-values derived from generalized estimating equations binomial model with an exchangeable correlation structure, accounting for within-patient clustering of bilateral incisors (inference at the patient level). Adjusted P-values were calculated using the Holm–Bonferroni correction.

*Significant P-values.

In the multivariate GEE model, which controlled for extraction status, treatment duration, and other covariates, greater U1 movement was the only independent predictor that remained statistically significant (OR = 1.76, 95% CI 1.21–2.56, P = 0.003; adjusted P = 0.030). IC height lost significance after adjustment (OR = 0.67, 95% CI 0.48–0.95, P = 0.022; adjusted P = 0.198) (Table 6).

In the linear mixed model, the magnitude of tooth movement and the initial root–IC distance emerged as the only independent predictors, after Holm–Bonferroni correction, for root-IC distance reduction (Supplementary Table S3). Greater U1 posterior movement was strongly associated with a larger reduction in root–IC distance (B = 0.40 mm per mm of movement; adjusted P < 0.001), and teeth that were initially farther from the canal also exhibited greater reductions (B = 0.135; adjusted P < 0.001). Treatment duration showed a small but statistically significant effect before correction (P = 0.044), but this association was not retained after multiplicity adjustment (adjusted P = 0.264). Importantly, extraction was not an independent predictor of root–IC distance change (B = 0.084; adjusted P = 0.395).

## 4 Discussion

This study provides comprehensive three-dimensional evidence of bone remodeling responses within the anterior maxilla, particularly around the incisive canal (IC), following orthodontic retraction with fixed appliances. Both linear and volumetric analyses demonstrated that tooth retraction, especially in extraction protocols, induces significant morphological and structural alterations in the IC and surrounding cortical bone, accompanied by reductions in root length and root–canal proximity.

The observed decreases in IC width and cortical bone width (CBW), particularly at the incisal (H1–H2) levels, indicate that extraction-driven retraction exerts greater mechanical loading on adjacent bone, leading to localized remodeling. These findings are consistent with previous reports linking extensive anterior movement and sustained force application to cortical thinning and canal adaptation (Chung et al., 2021; Yu et al., 2022). From a physiological standpoint, these alterations represent a regional bone adaptation process, in which osteoclastic resorption on the pressure side and osteoblastic apposition on the tension side collectively reshape the canal’s boundaries under continuous orthodontic stress. The increase in CBW-mid at H3 in non-extraction cases may reflect compensatory bone remodeling in areas of lower mechanical stimulus.

Volumetric and surface-area reductions further substantiate that IC changes extend beyond simple linear constriction, implying a three-dimensional remodeling response of osseous tissue to mechanical strain. The mixed-effects analysis strengthens the evidence that IC morphological adaptation is proximity-dependent rather than purely linear. The persistence of significance at H1 after Holm–Bonferroni correction supports a true biomechanical remodeling response near the canal entrance, where compressive and tensile forces concentrate during retraction. Such adaptive remodeling underscores the importance of evaluating bone physiology, not merely geometric boundaries, during orthodontic planning. In extraction cases, where retraction magnitudes are greater, cortical thinning and canal narrowing likely result from intensified bone turnover and localized stress concentration, elevating the risk of root proximity and resorption.

A significant decrease in U1 root–IC distance was observed post-treatment, with greater reductions in the extraction group across all measured levels. Specifically, reductions were 1.71 ± 0.78 mm at H1, 1.54 ± 1.09 mm at H2, and 1.55 ± 1.29 mm at H3, indicating a heightened likelihood of root–canal contact or invasion. These proximity changes reflect mechanical constraints within the alveolar housing and underscore the limited biological envelope for safe tooth movement. Root resorption was notably greater in the extraction group (2.02 ± 1.71 mm vs. 1.01 ± 0.74 mm, P = 0.005), correlating with canal contact and invasion and accompanied by greater volumetric and surface reductions in IC and U1 dimensions.

The progressive increase in root resorption from separation to invasion patterns confirms that closer U1–IC proximity represents a significant risk factor for apical shortening. The highest resorption observed at H2 aligns with the mid-root region’s reduced trabecular buffering capacity, suggesting that compressive stresses in this zone trigger intensified resorptive remodeling. This association reinforces the concept that when orthodontic forces exceed the physiological remodeling threshold, pathological resorption may occur as part of an unbalanced bone turnover response. The volumetric data further confirm that root–canal contact magnifies both dental and bony tissue remodeling, emphasizing the necessity of evaluating three-dimensional interactions rather than relying solely on linear distances.

The logistic regression findings further highlighted the biomechanical determinants of root–IC proximity. After accounting for patient-level clustering using Generalized Estimating Equations, univariate analysis identified several factors, including extraction protocol, treatment duration, U1 movement, and IC height, as significant predictors of root–IC contact or invasion. However, following Holm–Bonferroni correction and multivariate adjustment, only the magnitude of U1 movement remained an independent and statistically significant factor (OR = 1.76, 95% CI 1.21–2.56, P = 0.003; adjusted P = 0.030). This result underscores the predominant mechanical role of incisor displacement in driving root proximity and canal interaction, while anatomical variables such as IC height exert only secondary or context-dependent influence. These clustered-model outcomes reinforce that excessive retraction, rather than extraction status alone, governs the likelihood of canal contact and invasion.

From a clinical perspective, these findings underscore the importance of incorporating pre-treatment CBCT assessment, particularly in patients with tall or wide incisive canals or when planning large anterior retraction. Identification of high-risk canal anatomy should prompt careful torque control, anchorage reinforcement, and consideration of staged or light continuous forces to prevent excessive stress on the canal wall. In cases requiring substantial bodily movement, phased retraction mechanics or root-uprighting strategies may further reduce the likelihood of canal contact and treatment-related resorption.

The linear mixed model analysis further clarified the determinants of continuous changes in root–IC distance. After accounting for within-patient clustering and correcting for multiplicity, only the initial root–IC distance and the magnitude of U1 movement remained significant predictors of reduction magnitude. Teeth that began farther from the canal and experienced greater posterior displacement demonstrated proportionally larger reductions, highlighting the biomechanical nature of root–canal approximation. In contrast, extraction protocol showed no independent effect once displacement was controlled, and the model-estimated group difference (1.37 mm vs. 1.28 mm) was small and not statistically reliable. These findings reinforce that anatomical starting position and the actual degree of incisor translation, rather than extraction choice itself, primarily govern how much the root apex migrates toward the incisive canal.

From a physiological perspective, these findings highlight the incisive canal’s role not merely as an anatomical landmark but as a dynamic osseous structure capable of remodeling in response to orthodontic loading. CBCT-based volumetric analysis provides a unique window into this adaptation, allowing quantification of both geometric and biological changes. Identifying high-risk patterns, such as taller canals, narrower U1–IC spacing, or high retraction magnitude, can help clinicians anticipate bone adaptation limits and optimize biomechanical strategies.

The present findings provide novel insight into the craniofacial bone’s adaptive response to orthodontic loading. Changes in incisive canal morphology and surrounding cortical structures reflect site-specific remodeling driven by mechanical stress redistribution during tooth retraction. Extraction-based retraction, involving greater displacement and force magnitude, elicits more pronounced bone turnover and canal adaptation, emphasizing the dynamic nature of craniofacial bone physiology. Understanding these remodeling patterns enhances our ability to predict biological limits, optimize force application, and promote tissue preservation in orthodontic biomechanics.

It should be noted that the present study evaluated post-treatment changes immediately after debonding. Since bone and canal remodeling may continue during the retention phase, the current results likely represent early-stage adaptive responses to mechanical loading rather than long-term stabilization. Future longitudinal studies incorporating post-retention CBCT imaging could better characterize these ongoing remodeling processes.

In summary, clinicians should consider incisive canal morphology and its remodeling potential when planning retraction mechanics. Incorporating 3D CBCT evaluation into treatment planning, especially for extraction cases with large planned retraction or tall ICs, can help identify anatomical limitations, tailor force application, and reduce the risk of root resorption and neurovascular compromise.

The present study has several limitations. Its retrospective design and single-country sample may limit generalizability across populations and treatment protocols. All cases were treated with a single fixed-appliance protocol, which may not reflect outcomes achievable with other mechanics. While bilateral measurements were averaged at the patient level for continuous linear and volumetric comparisons (Tables 1, 2) to preserve statistical independence, proximity patterns and incisor-level outcomes (Tables 3–6) were analyzed per tooth to accurately capture asymmetrical root–IC interactions. Although analysis at the tooth level introduces within-patient clustering, this limitation was addressed by using appropriate clustered models, GEE for categorical outcomes and Linear Mixed Models for continuous outcomes, thereby providing valid standard errors and inference at the patient level. Nonetheless, some residual within-subject dependence cannot be completely excluded. Case selection was restricted to subjects exhibiting primarily bodily movement of the maxillary incisors, which may introduce selection bias and limit applicability to tipping or torque movements. The CBCT voxel size (0.3 mm) may not capture subtle microstructural changes and is susceptible to partial-volume effects. Neuro-sensory outcomes, such as nasopalatine discomfort or paresthesia, were not assessed, limiting insight into functional implications. Finally, follow-up was confined to the immediate post-treatment (T2) stage; hence, long-term bone remodeling and stability remain unverified.

Future studies should adopt prospective, multi-center designs with extended post-retention imaging, include diverse movement patterns, and incorporate patient-reported outcomes to better delineate biological adaptation and clinical relevance.

## 5 Conclusion

Orthodontic retraction of maxillary central incisors reduces the root–incisive canal distance, with an adjusted mean reduction of approximately 1.33 mm. Critically, the magnitude of tooth movement, rather than extraction status, is the primary determinant of canal approximation. Each millimeter of U1 retraction resulted in an additional 0.40 mm decrease in root–IC distance and 1.76-fold higher odds of root–canal contact or invasion (95% CI, 1.21–2.56). Root resorption was also proximity-dependent, with incisors exhibiting contact or invasion showing 0.38 mm greater apical shortening than those maintaining separation (95% CI, 0.08–0.69 mm). These findings highlight the need for careful CBCT-based assessment and controlled force application in cases requiring substantial anterior retraction.

## Data Availability

The original contributions presented in the study are included in the article/Supplementary Material, further inquiries can be directed to the corresponding author.
