# Supplementary material for: Comparison of incisive canal remodeling and root resorption in extraction vs. non-extraction fixed orthodontic retraction: a CBCT study
Source: Front Physiol. 2025 Dec 18;16:1726454. doi: 10.3389/fphys.2025.1726454 (PMC12756103; doi:10.3389/fphys.2025.1726454)
Supplement: Supplementary file 1 [file Table1.docx]

**Supplementary Material 2**

**Table 1.** Demographic characteristics and baseline ABO discrepancy index scores.

| **Item** | **Non-extraction (n =44)** | **Extraction (n =42)** | **P value** |
| --- | --- | --- | --- |
| **Male, n (%)** | 18 (40.91) | 18 (42.86) | 1.000 |
| **Female, n (%)** | 26 (59.09) | 24 (57.14) |  |
| **Age, y** | 22.16±6.35 | 22.41±5.09 | 0. 838 |
| **Treatment duration, y** | 2.72±0.96 | 3.19±1.14 | 0.097 |
| **U1 move, mm** | −1.60±1.68 | −3.25±1.72 | **<0.001*** |
| **SNA (°)** | 82.82±3.14 | 82.91±3.12 | 0.933 |
| **SNB (°)** | 77.61±3.58 | 77.56±3.84 | 0.514 |
| **U1-SN (°)** | 100.94±8.03 | 106.40±14.30 | **<0.001*** |
| **ABO scores, points** |  |  |  |
| **Overjet** | 0.98±1.28 | 1.67±1.28 | **0.010*** |
| **Overbite** | 0.93±1.11 | 1.50±1.25 | **0.026*** |
| **Anterior open bite** | 0±0 | 1.14±3.89 | 0.053 |
| **Posterior open bite** | 0±0 | 0±0 | 1.000 |
| **Crowding** | 0.5±0.21 | 1.33±0.48 | **0.001*** |
| **Occlusion** | 2.82±1.99 | 2.86±1.47 | 0.953 |
| **Posterior crossbite** | 0.55±2.04 | 0±0 | 0.242 |
| **ANB** | 1.29±2.16 | 1.17±2.01 | 0.711 |
| **SN-GoGn** | 1.68±2.18 | 1.65±2.21 | 0.652 |
| **IMPA** | 4.52±6.04 | 3.43±4.52 | 0.324 |
| **Total ABO score** | 12.81±8.93 | 13.03±5.70 | 0.476 |
| * Significant P-values; continuous variables are reported as mean ± standard deviation in years (y), millimeters (mm), points, or degrees (°); sex is reported by frequency and rate, n (%). | | | |

Table 2. Linear mixed model analysis comparing changes (T2–T1) in incisive canal and central incisor volumes and surface areas according to root–IC proximity patterns.

| **Root-IC proximity** | **IC volume, mm³** | | | | **IC area, mm²** | | | | **U1 volume, mm³** | | | | **U1 area, mm²** | | | |
| --- | --- | --- | --- | --- | --- | --- | --- | --- | --- | --- | --- | --- | --- | --- | --- | --- |
|  | **Adjust mean (95% CI)** | **SE** | **P** | **Adj. P** | **Adjust mean (95% CI)** | **SE** | **P** | **Adj. P** | **Adjust mean (95% CI)** | **SE** | **P** | **Adj. P** | **Adjust mean (95% CI)** | **SE** | **P** | **Adj. P** |
| **H1** |  |  |  |  |  |  |  |  |  |  |  |  |  |  |  |  |
| Separation | 22.46  (13.44-31.48) | 4.55 | **0.003*** | **0.009*** | 17.73  (6.89-28.57) | 5.47 | **0.007*** | **0.021*** | 12.96  (1.78-27.70) | 7.36 | NS | NS | 6.97  (1.15-12.79) | 2.93 | **0.044*** | NS |
| Approximation | 24.48  (15.38-33.58) | 4.60 | **0.009*** | **0.018*** | 22.30  (14.88-29.71) | 3.74 | **0.009*** | **0.021*** | 20.77  (1.12-40.42) | 9.86 | NS | NS | 12.03  (9.54-14.53) | 1.26 | NS | NS |
| Contact | 23.22  (9.99-36.45) | 6.67 | **0.036*** | **0.036*** | 24.88  (17.39-32.37) | 3.78 | **0.042*** | **0.042*** | 25.43  (17.88-32.97) | 3.79 | NS | NS | 12.76  (8.62-16.90) | 2.08 | NS | NS |
| Invasion (ref) | 38.37  (33.26-43.49) | 2.59 | – | – | 33.83  (29.58-38.08) | 2.15 | – | – | 26.50  (10.09-42.91) | 8.24 | – | – | 14.25  (10.19-18.31) | 2.04 | – | – |
| **H2** |  |  |  |  |  |  |  |  |  |  |  |  |  |  |  |  |
| Separation | 25.85  (12.88-38.82) | 6.54 | NS | NS | 22.76  (12.24-33.29) | 5.33 | NS | NS | 18.61  (9.96-27.25) | 4.35 | NS | NS | 8.78  (1.74-15.81) | 3.48 | NS | NS |
| Approximation | 25.40  (12.84-37.96) | 6.36 | NS | NS | 26.44  (15.52-37.36) | 5.51 | NS | NS | 18.82  (14.45-23.20) | 2.21 | NS | NS | 11.17  (7.67-14.68) | 1.74 | NS | NS |
| Contact | 25.74  (15.94-35.56) | 4.95 | NS | NS | 27.20  (18.93-35.46) | 4.17 | NS | NS | 19.09  (7.64-30.53) | 5.77 | NS | NS | 12.87  (3.38-22.35) | 4.71 | NS | NS |
| Invasion (ref) | 34.91  (30.04-39.77) | 2.47 | – | – | 30.49  (26.41-34.57) | 2.06 | – | – | 23.99  (12.65-35.32) | 5.73 | – | – | 17.59  (10.85-24.33) | 3.37 | – | – |
| **H3** |  |  |  |  |  |  |  |  |  |  |  |  |  |  |  |  |
| Separation | 22.60  (13.34-31.86) | 4.68 | **0.032*** | NS | 21.05  (13.38-28.71) | 3.87 | NS | NS | 13.28  (9.33-17.25) | 1.99 | NS | NS | 9.41 (7.55-11.88) | 1.09 | NS | NS |
| Approximation | 30.88  (25.96-35.81) | 2.49 | 0.088 | NS | 27.56  (23.49-31.64) | 2.06 | NS | NS | 16.14  (8.70-23.59) | 3.75 | NS | NS | 14.23 (10.15-18.30) | 2.05 | NS | NS |
| Contact | 40.55  (31.77-49.33) | 4.44 | 0.273 | NS | 38.91  (31.65-46.16) | 3.67 | NS | NS | 33.51  (26.40-40.63) | 3.59 | NS | NS | 15.98 (12.07-19.89) | 1.97 | NS | NS |
| Invasion (ref) | 58.81  (27.04-90.58) | 15.93 | – | – | 41.70  (15.34-68.06) | 13.25 | – | – | 26.20  (3.30-49.10) | 11.49 | – | – | 16.21 (4.21-28.21) | 6.02 | – | – |
| Data are adjusted means in square millimeters, mm² or cubic millimeters, mm^3^ with (95% CI, confidence interval) from Linear Mixed Models with random intercept for patient, accounting for clustering of bilateral measurements. Adjusted P values derived using Holm–Bonferroni correction across proximity patterns per level. * Significant *P*-values (P < 0.05) indicate greater reduction relative to invasion (reference). NS, not significant. | | | | | | | | | | | | | | | | |

**Table 3.** Linear mixed model analysis identifying predictors of root–IC distance reduction (mm) (T2–T1).

| **Factor** | **Estimate (B)** | **95% CI** | **SE** | **P-value** | **Adjusted**  **P-value** |
| --- | --- | --- | --- | --- | --- |
| **Extraction, n (%) ^a^** | 0.084 | −0.009, 0.178 | 0.047 | 0.079 | 0.395 |
| **Sex, n (%) ^b^** | 0.052 | −0.036, 0.139 | 0.044 | 0.245 | 0.980 |
| **Age, y** | −0.001 | −0.009, 0.007 | 0.004 | 0.779 | 1.000 |
| **Treatment duration, y** | −0.042 | −0.084, −0.001 | 0.020 | **0.044*** | 0.264 |
| **U1 movement, mm** | 0.401 | 0.367, 0.430 | 0.016 | **<0.001*** | **<0.001*** |
| **IC height, mm** | −0.003 | −0.040, 0.035 | 0.018 | 0.894 | 1.000 |
| **Root-IC distance, mm** | 0.135 | 0.075, 0.194 | 0.030 | **<0.001*** | **<0.001*** |
| **ABO index score, point** | 0.003 | −0.002, 0.009 | 0.003 | 0.258 | 0.980 |
| Continuous variables are measured in years (y), millimeters (mm), or points; extraction and sex are reported by frequency and rate, n (%).  CI, confidence interval; SE, standard error. a, non-extraction is the reference; b, female is the reference.  P-values derived from Linear Mixed Model with random intercept for patient, accounting for clustering of bilateral measurements (inference at patient level); Adjusted P values were calculated using the Holm–Bonferroni correction. * Significant *P*-values | | | | | |
