# Supplementary material for: Comparison of incisive canal remodeling and root resorption in extraction vs. non-extraction fixed orthodontic retraction: a CBCT study
Source: Front Physiol. 2025 Dec 18;16:1726454. doi: 10.3389/fphys.2025.1726454 (PMC12756103; doi:10.3389/fphys.2025.1726454)
Supplement: Supplementary file 2 [file DataSheet1.pdf]

Supplementary Material 1

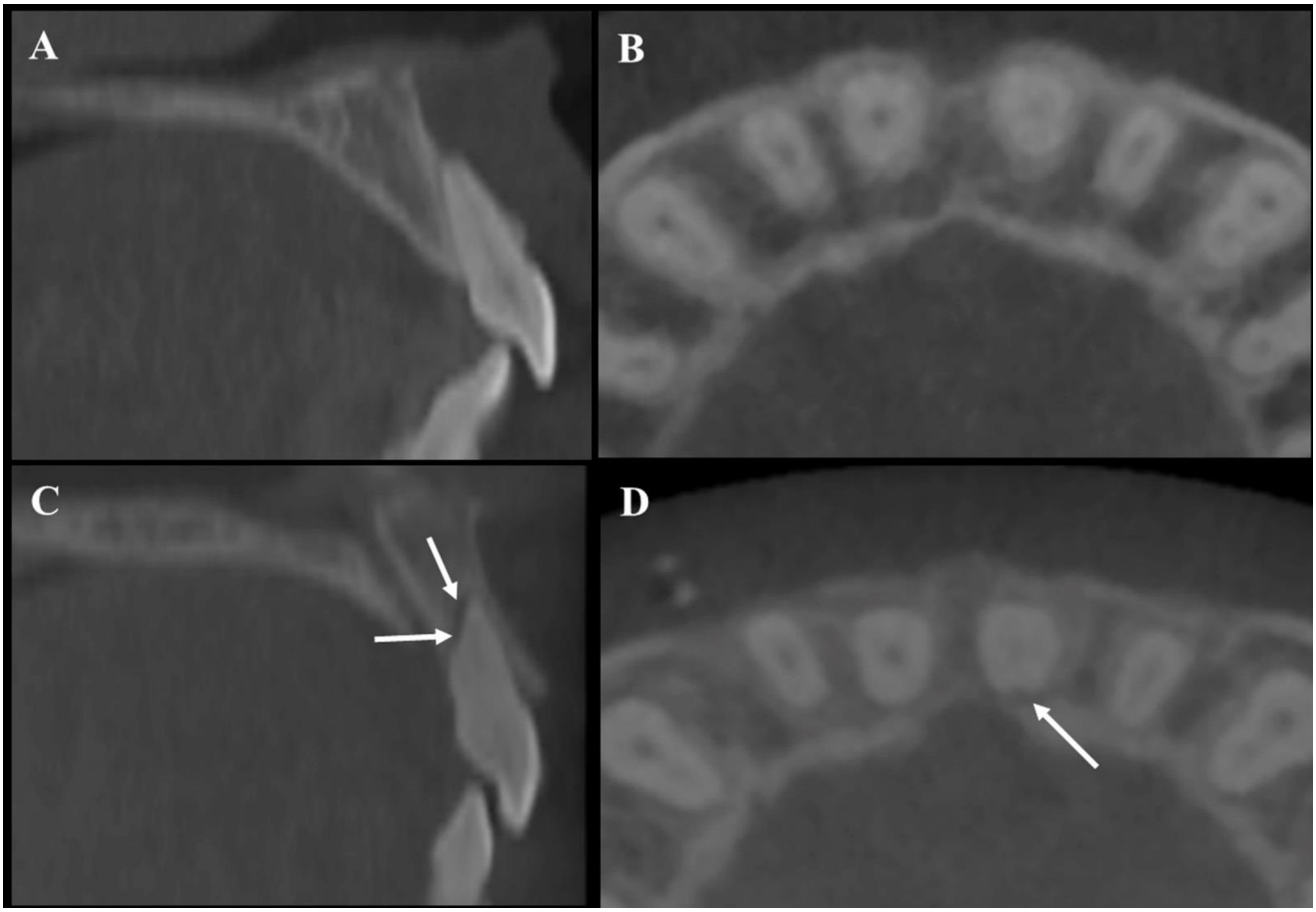

Fig. 1. Examples of post-treatment root resorption (arrows): Preoperative scans (A, B); Postoperative scans showing apical root resorption (C) and horizontal root resorption (D).

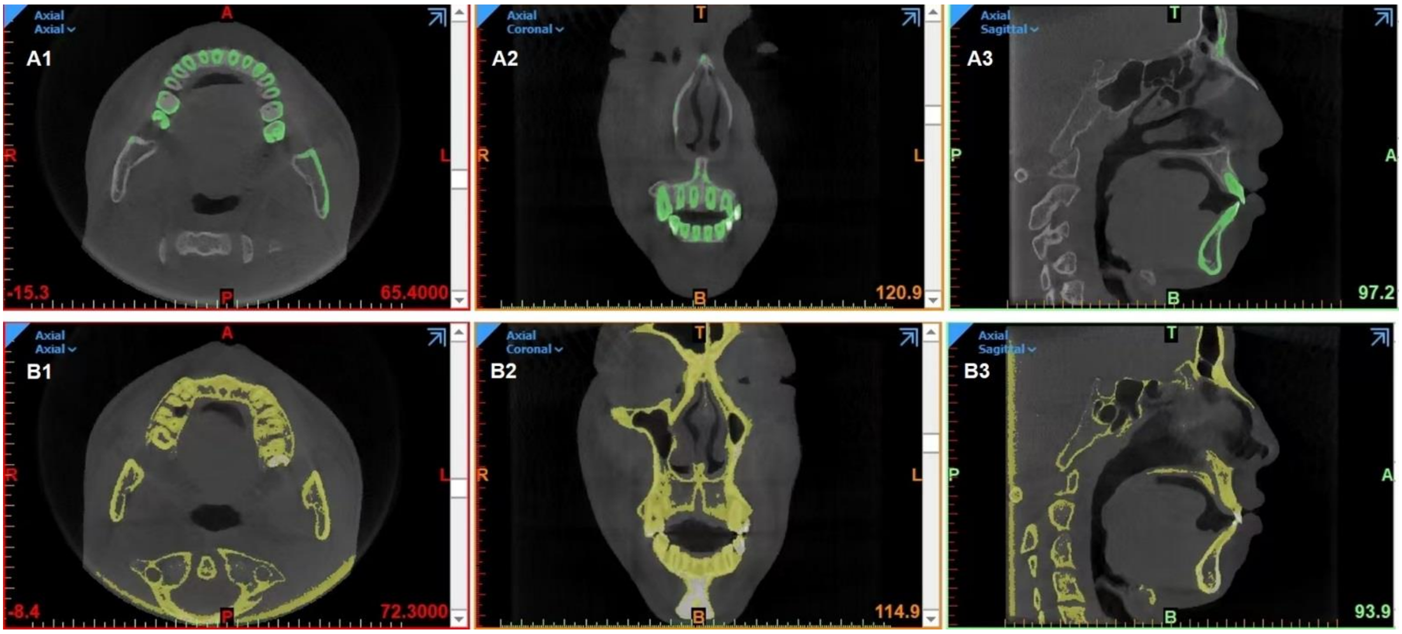

Fig. 2. Mask–image overlays for volumetric analysis of the maxillary central incisor (A1-A3) and incisive canal (B1-B3).

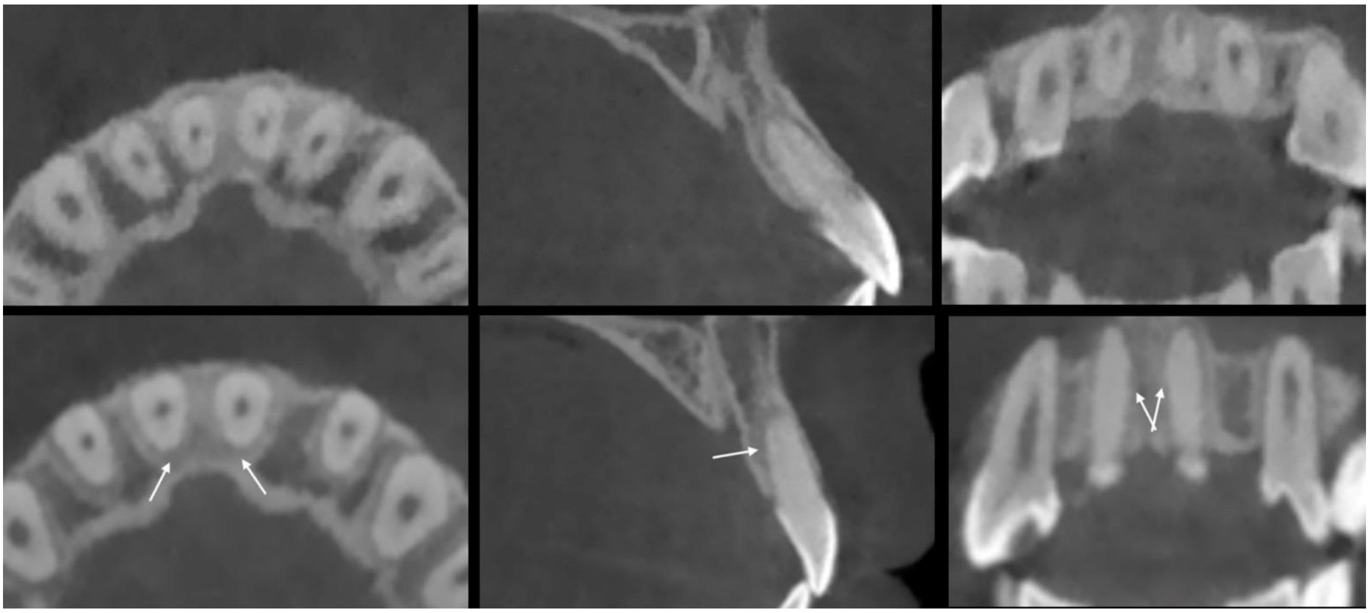

Fig. 3. Representative example of the root-IC separation pattern in axial, sagittal, and coronal views. Top, preoperative scan. Bottom, postoperative scan showing root-IC separation (arrows).

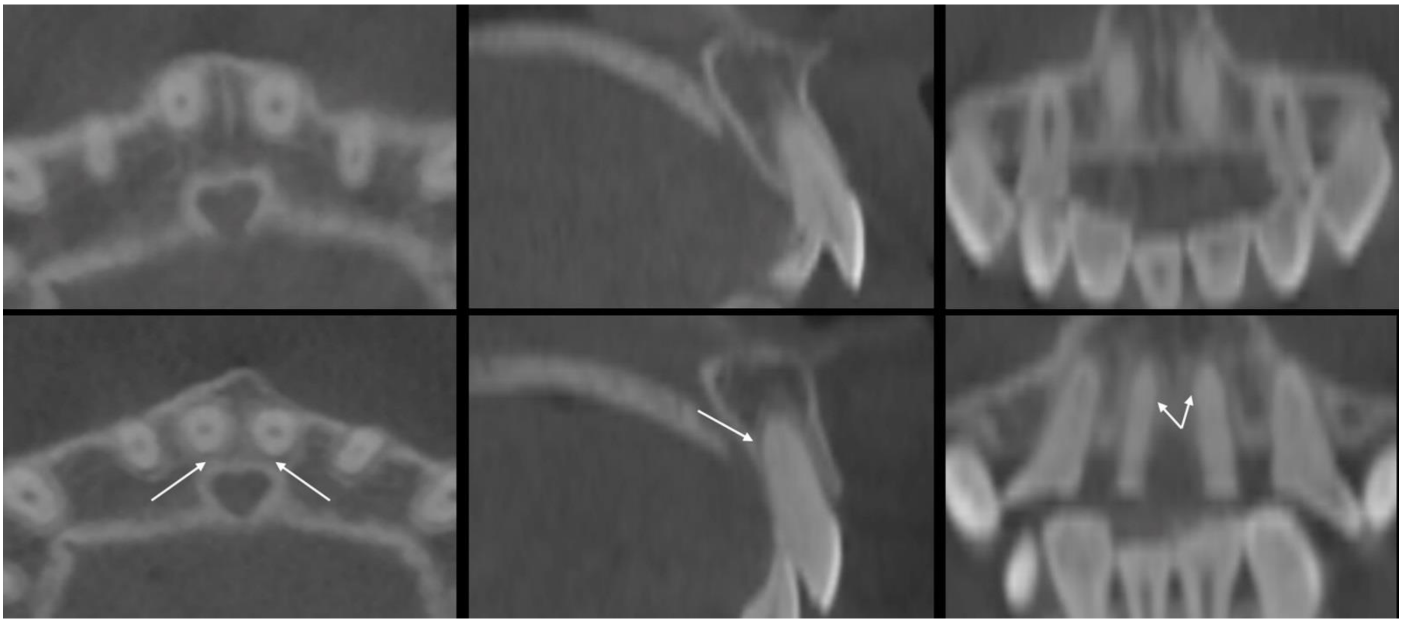

Fig. 4. Representative example of the root-IC approximation pattern in axial, sagittal, and coronal views.

Top, preoperative scan. Bottom, postoperative scan showing root-IC approximation (arrows).

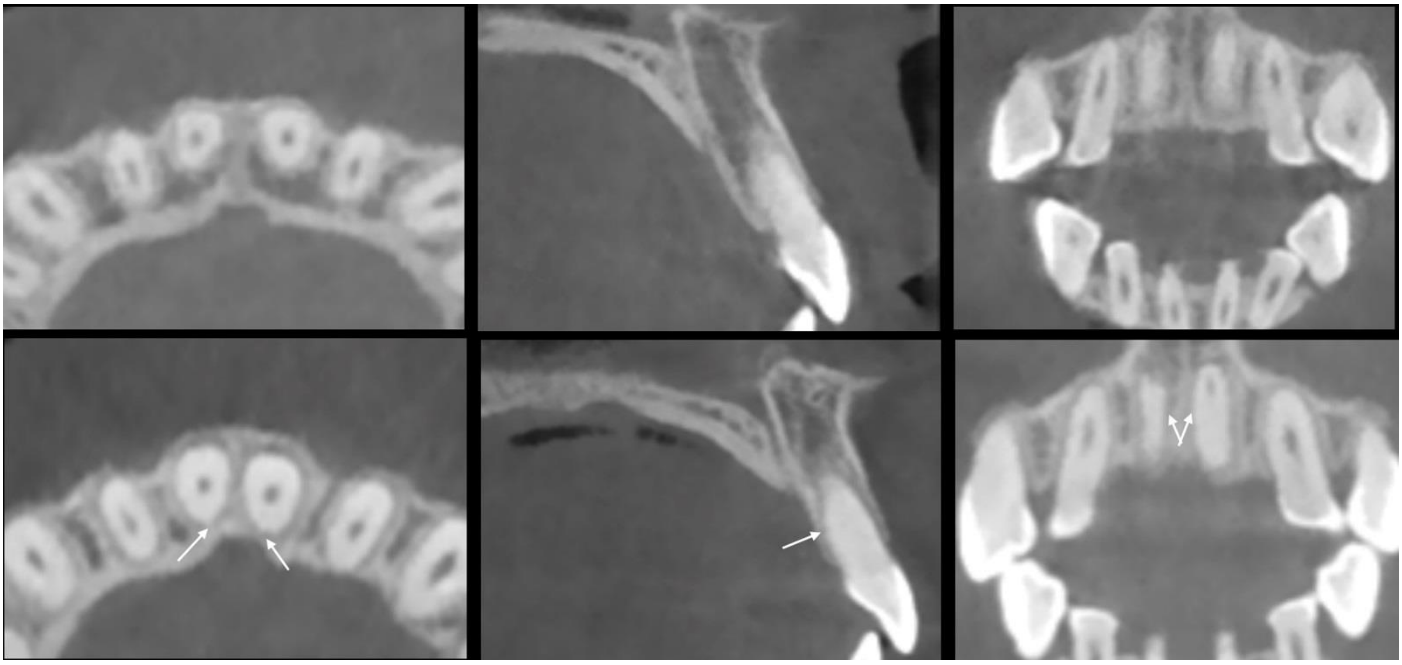

Fig. 5. Representative example of the root-IC contact pattern in axial, sagittal, and coronal views. Top, preoperative scan. Bottom, postoperative scan showing root-IC contact (arrows).

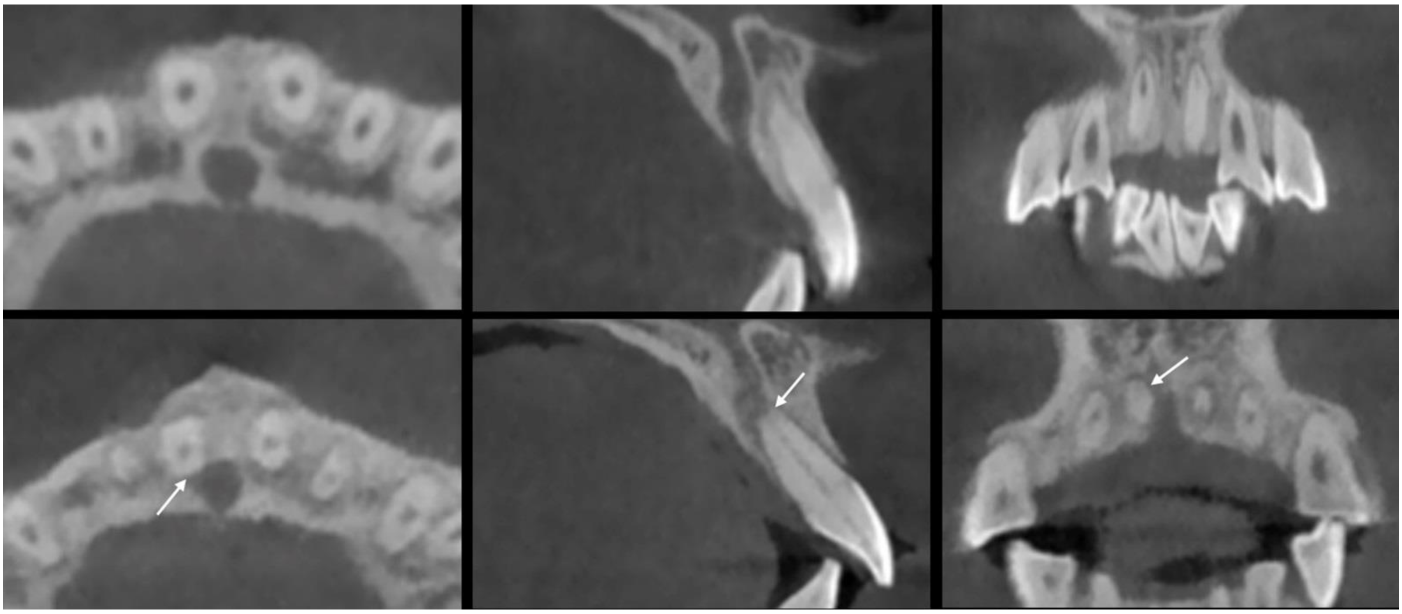

Fig. 6. Representative example of the root-IC invasion pattern in axial, sagittal, and coronal views. Top, preoperative scan. Bottom, postoperative scan showing root-IC invasion (arrows).
